# Canopy Design Drives Photosynthetic Performance, Light Environment, and Fruit Quality in Peach (*Prunus persica* L. Batsch)

**DOI:** 10.3390/plants15010029

**Published:** 2025-12-21

**Authors:** Ioannis Chatzieffraimidis, Dimos Stouris, Marina-Rafailia Kyrou, Fokion Papathanasiou, Evangelos Karagiannis

**Affiliations:** Department of Agriculture, School of Agricultural Sciences, University of Western Macedonia, 53100 Florina, Greece

**Keywords:** peach, *Prunus persica*, training systems, canopy architecture, photosynthesis, leaf area index (LAI), fruit quality

## Abstract

Training system selection critically influences peach orchard productivity through its effects on canopy light environment, physiological responses, and fruit quality. This study evaluated two contrasting training systems: a 2D planar fruiting wall system (Four-Axis, 1020 trees ha^−1^) versus a 3D Quad-V system (590 trees ha^−1^) using two peach cultivars, fresh table ‘Platibelle’ and clingstone ‘Mirel’ in Central Macedonia, Greece. Comprehensive physiological measurements including leaf gas exchange, chlorophyll fluorescence, and fruit quality parameters were assessed across two canopy zones (lower 0–1.2 m vs. upper 1.8–3.3 m) during the 2023 and 2024 growing seasons. Results demonstrated that the 2D system achieved superior leaf area index (LAI), but lower light interception, leading to enhanced photosynthetic performance with 15–20% higher net photosynthetic rates and improved water-use efficiency compared to the 3D system. Notably, the photosynthetic apparatus of fruiting wall trees maintained significantly greater efficiency (6.26 μmol CO_2_ m^−2^ s^−1^) in the lower canopy zone than in Quad-V trees (3.6 μmol CO_2_ m^−2^ s^−1^), indicating a more uniform and functional light environment. The 2D system produced fruits with improved flesh firmness and color development in ‘Mirel’, while higher dry matter in ‘Platibelle’. Correlation analysis revealed that Four-Axis trees enhanced the interdependence among thermal, gas exchange, and compositional traits, reflecting a shift from morphology-driven to metabolically integrated canopy function. In terms of yield, fruiting walls achieved higher efficiency and total production (Mt ha^−1^) in ‘Mirel’, supporting their adoption to enhance productivity and peach fruit quality in Mediterranean conditions.

## 1. Introduction

Modern peach (*Prunus persica* L. Batsch) cultivation, is one of the most important fruit crops of the Mediterranean basin, yet its production increasingly faces challenges linked to market demand for improved fruit quality and orchard efficiency, rising labor costs, and reduced availability of skilled workers [1,2,3,4,5]. A wide range of preharvest factors are known to affect fruit development and quality, including crop load [6,7], as well as canopy design, which determines fruit position within the tree and the prevailing light environment [8,9]. Over the past 15 years, commercial peach orchards worldwide have been gradually shifting from traditional low-density training systems with complex three-dimensional (3D) canopies toward intensified orchard designs characterized by higher planting densities and simplified, two-dimensional (2D) planar structures [3,4,10,11]. While traditional 3D canopies can achieve high yields, their structural complexity drives production higher into the canopy, requires more labor for pruning, thinning, and harvesting, and results in heterogeneous light distribution, which compromises both orchard efficiency and fruit uniformity, limiting their economic sustainability [12,13]. The relative advantages of these systems remain strongly influenced by cultivar traits and growing environment. Consequently, there has been a global trend toward intensification through higher-density plantings combined with simplified canopy architectures designed to optimize light interception and distribution while facilitating mechanization [4,11,14,15].

Training systems fundamentally influence orchard productivity by shaping how light is captured and distributed within the canopy [16,17], thereby determining photosynthetic efficiency and fruit development [18,19,20]. The relationship between light interception and fruit tree productivity is well established, with earlier studies demonstrating linear correlations between intercepted photosynthetically active radiation (PAR) and net carbon assimilation [16,21,22,23]. Yet, this relationship tends to weaken when interception exceeds about half of the incoming radiation, underscoring that the spatial distribution of light within the canopy can be as important as the total amount intercepted [24,25], and this principle is repeatedly demonstrated across high-density fruit systems, including apples under Tall Spindle and Vertical Axis systems [11].

Two-dimensional fruiting wall systems have gained prominence due to their potential for improved light distribution, reduced canopy complexity, and enhanced mechanization compatibility [2,26], and show parallels with 2D planar systems used in pears, apples, and olives, which improve uniformity and reduce canopy complexity [3,4,27]. These systems typically employ higher planting densities (1000–3000 trees ha^−1^) with simplified canopy structures featuring single or multiple vertical leaders arranged in planar configurations, yet the optimal planting density is not clear [1], while 3D systems such as the Quad-V maintain complex canopy architectures with four main scaffolds per tree at lower densities (500–1000 trees ha^−1^), requiring more intensive management but potentially offering greater individual tree productivity [10,20,28,29]. Physiological performance varies across training systems, with differences reported in photosynthetic capacity, water-use efficiency, and tolerance to stress conditions [11,30,31]. Canopy position effects are particularly pronounced in complex 3D systems, where interior shading can significantly reduce photosynthetic capacity and fruit quality in lower canopy zones [26,32,33]. Light availability within the canopy directly influences chlorophyll fluorescence parameters, stomatal conductance, and photosynthetic electron transport efficiency, ultimately affecting carbon assimilation and fruit development [18,34,35].

Fruit quality in peach is intimately linked to canopy light environment, with traits such as soluble solids concentration, flesh firmness, and color development responding positively to increased light exposure [8,17], and similar canopy-light-dependent quality differences are observed in apples and olives grown in high-density planar systems [4,11]. Canopy position effects on fruit quality have been well documented, with upper canopy fruits typically exhibiting advanced maturity, higher dry matter content, and superior color development compared to shaded positions [9]. Beyond light exposure, the leaf-to-fruit ratio emerges as a critical factor influencing fruit quality, with optimal ratios varying according to training system, cultivar, and environmental conditions [6,7,28].

Greece ranks among the leading EU peach-producing countries, and the region of Central Macedonia represents its most important production hub [36]. Climate change compounds production challenges in the region; higher summer temperatures, more frequent water limitations, and variable radiation environments demand orchard designs that secure consistent productivity and fruit quality [2] and reflect broader challenges where optimized planting densities improve efficiency under climate stress [4,37,38].

The objective of this study was to provide a comprehensive comparison of 2D planar fruiting wall versus 3D Quad-V training systems in peach, focusing on (i) canopy light environment and distribution patterns, (ii) physiological responses including leaf gas exchange and chlorophyll fluorescence, (iii) fruit quality characteristics across canopy zones, and (iv) correlations between leaf area index (LAI), training systems, and fruit quality traits. The novelty of this study lies in the integrated evaluation of canopy light environment, physiology, yield efficiency, and fruit physicochemical traits, as well as the simultaneous assessment of the interactions between training system, canopy zone, and crop load under open-field commercial conditions.

## 2. Results and Discussion

**Figure 1:**
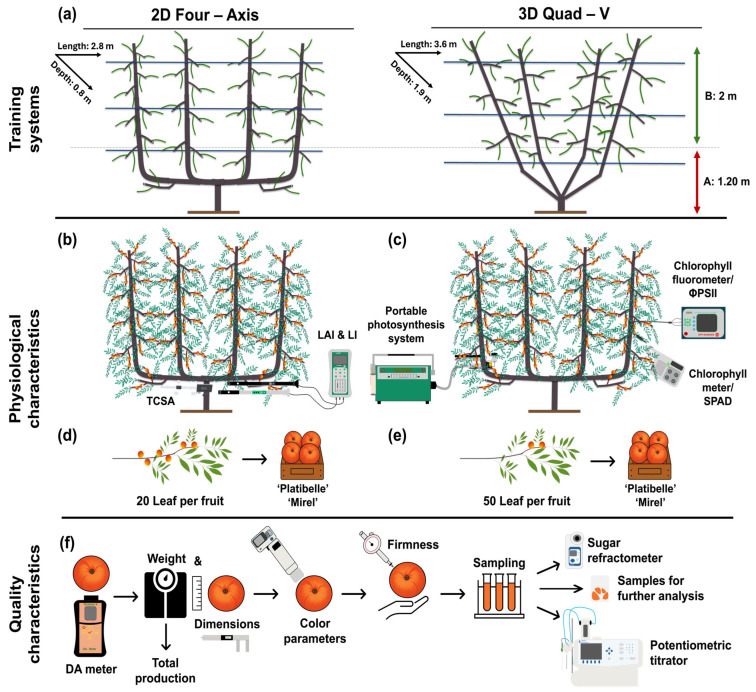
Experimental design to assess the effects of two training systems on peach tree physiology, light environment, yield, and fruit quality. (**a**) Trees trained either to a planar two-dimensional (2D) fruiting wall system or to a three-dimensional (3D) Quad-V system; within each system, canopy zones were stratified into upper (green arrow) and lower (red arrow) layers for physiological and fruit quality assessments. (**b**) Morphological parameters (trunk cross-sectional area, TCSA, tree height) and light environment variables (leaf area index, LAI; photosynthetically active radiation, PAR; light interception, LI; light distribution index, LDI) were measured at multiple canopy heights. (**c**) Physiological performance was accessed by leaf gas exchange, chlorophyll fluorescence, and chlorophyll concentration. (**d**,**e**) At harvest, fruits sampled by canopy zone and leaf-to-fruit ratio (20 vs. 50 leaves fruit^−1^), (**f**) yield per tree and orchard productivity (t ha^−1^) were determined, and equal maturity level based on DA-meter index were evaluated for physicochemical quality attributes, including firmness, soluble solids content, and dry matter concentration. The schematic illustrates the overall experimental layout and the methodology used for each measurement category, enabling the integration of canopy architecture, physiology, productivity, and fruit quality traits.

The effects of canopy architecture on light interception, physiological performance, and fruit quality were evaluated in two training systems (a planar two-dimensional fruiting wall (2D) and a three-dimensional Quad-V system (3D) (Figure 1a). The experimental design integrated multilevel canopy assessments to link tree vigor, light environment, photosynthetic function, and fruit quality development across two consecutive growing seasons (2023–2024). Morphological traits including trunk cross-sectional area (TCSA) and tree height were measured to quantify tree vigor (Figure 1b). The canopy light environment was characterized through measurements of photosynthetically active radiation (PAR) and the derived parameters of light interception (LI), light availability, and the light distribution index (LDI), while LAI was measured at four canopy heights to describe vertical foliage distribution (Figure 1b). Physiological measurements, including leaf gas exchange, chlorophyll fluorescence, and chlorophyll concentration, were conducted on leaves sampled from the lower (0–1.2 m) and upper (1.8–3.0 m) canopy zones (Figure 1c). At harvest, yield per tree and orchard productivity (t ha^−1^) were determined, and fruit were further sampled by canopy zone and leaf-to-fruit ratio classes (20 vs. 50 leaves fruit^−1^) for quality analysis (Figure 1d–f).

### 2.1. Leaf Area, Light Environment, and Canopy Structure

**Figure 2:**
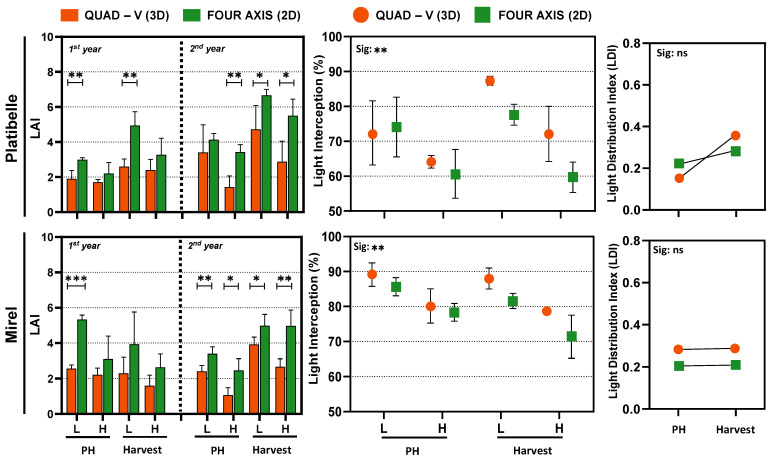
Characterization of canopy zones, structure, and light environment in ‘Platibelle’ and ‘Mirel’ peach trees trained to two canopy architectures, a planar two-dimensional (2D, green) and a three-dimensional (3D, orange) system, during the 2023 and 2024 growing seasons. Average leaf area index (LAI), light interception (LI), and light distribution index (LDI) were measured non-destructively at pit hardening (PH) and harvest stages across lower (L) and upper (H) canopy zones. Points and bars represent mean ± SE of four replicates per canopy zone, training system, and cultivar; asterisks indicate significant differences between canopy zones or training systems (* *p* < 0.05; ** *p* < 0.01; *** *p* < 0.001; ns = not significant) according to Duncan’s multiple range test (*p* ≤ 0.05). Student’s *t*-test was applied after ANOVA to compare training systems within each canopy zone for LAI.

Across 2023–2024, LAI was consistently higher in the 2D system than in Quad-V. In ‘Platibelle’, LAI ranged 2.21–6.66 in 2D vs. 1.43–4.73 in Quad-V; in ‘Mirel’, 2.47–5.34 vs. 1.07–3.93 (*p* < 0.01–0.001), reflecting more efficient leaf deployment per ground area in planar canopies (Figure 2). Similar trends have been reported in peach and pear orchards, where high-density planar systems typically support higher LAI than traditional training systems [1,39].

Despite higher LAI in 2D (Figure 2), per-tree LI was greater in Quad-V (Platibelle: 64.10–87.35% vs. 59.65–77.61%; Mirel: 78.69–89.14% vs. 71.38–85.67%), consistent with the greater light interception of larger 3D canopies due to volume and leaf density [40].

Although numerical differences were observed in LDI between training systems, these differences were not statistically significant (Figure 2). Managing the light environment through canopy structure and daily light exposure profiles enhances photochemistry, reduces asymmetries between sunlit and shaded zones, and improves the internal energy balance of foliage [16,41]. This supports the idea that uniform LDI, rather than maximum interception (LI), is the more critical factor for sustainable photosynthetic performance [18].

While 3D Quad-V canopies maximize absolute interception, they concentrate irradiance in the outer layers and exacerbate vertical light stratification. In contrast, 2D ‘fruiting wall’ systems, despite slightly lower total interception, improve internal light penetration and uniformity, which are increasingly recognized as critical for sustained photosynthetic efficiency, fruit quality, and long-term orchard productivity [1,14,39]. The narrow canopy width (60–80 cm) of fruiting walls further enhances penetration (Figure S1), aligning with recommendations for modern high-density orchards [9].

### 2.2. Physiological Responses to Training Systems

#### 2.2.1. Leaf Gas Exchange Performance

**Figure 3:**
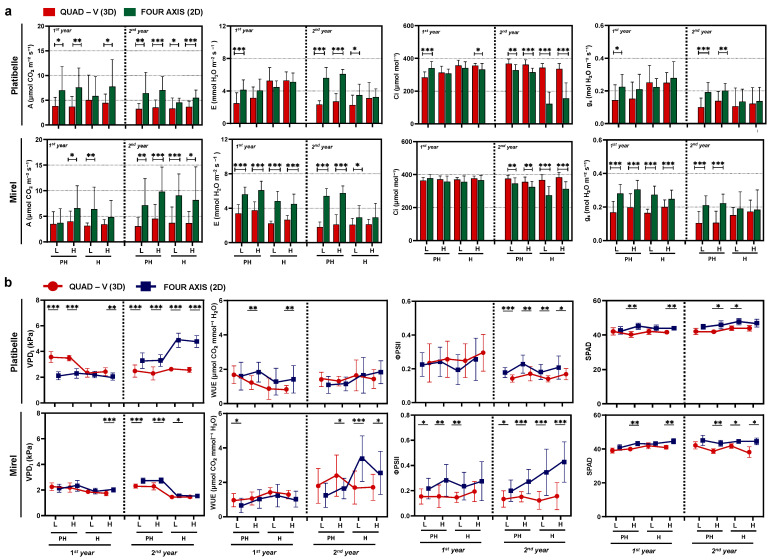
Physiological and photosynthetic performance of ‘Platibelle’ and ‘Mirel’ peach trees trained to two canopy architectures, a 2D fruiting wall and a three-dimensional (3D) during the 2023 and 2024 growing seasons. Parameters include (**a**) net photosynthetic rate (A), stomatal conductance (g_s_), transpiration rate (E), intercellular CO_2_ concentration (C_i_), and (**b**) leaf vapor pressure deficit (VPD_l_), water-use efficiency (WUE = A/E), photosystem II quantum yield (ΦPSII), and chlorophyll concentration (SPAD). Measurements were conducted at pit hardening (PH) and harvest (H) across lower (L) and upper (H) canopy zones. Symbols and bars represent mean ± SE of four replicates per canopy zone, training system, and cultivar; asterisks indicate significant differences between canopy zones and training systems (* *p* < 0.05; ** *p* < 0.01; *** *p* < 0.001; ns = not significant) according to Duncan’s multiple range test (*p* ≤ 0.05). Student’s *t*-test was applied after ANOVA to compare training systems within each canopy zone.

Significant differences in gas exchange parameters were observed between training systems, phenological stages, and canopy zones across both years (Figure 3a). The 2D system consistently showed higher net photosynthetic rates. In ‘Platibelle’, values ranged 4.5–7.6 μmol CO_2_ m^−2^ s^−1^ in 2D vs. 3.3–5.1 in Quad-V; in ‘Mirel’, 3.7–9.8 vs. 3.1–4.5. This represents approximately 15–20% improvement in photosynthetic performance, attributable to greater light availability within the canopy. When averaged across stages and years, assimilation was about 70% higher in 2D (58% in ‘Platibelle’ and 90% in ‘Mirel’) than in Quad-V, underscoring the strong influence of improved canopy light distribution. Enhanced assimilation in planar systems reflects improved light penetration and reduced self-shading, a key advantage also reported in narrow orchard systems of apple and peach [14,40]. Previous work shows that as soon as 50–60% of incoming radiation is intercepted, gains in total photosynthesis plateau and distribution quality become the limiting factor [22,42]. Thus, the stronger performance of 2D canopies highlights the central role of light distribution over total interception. The present results provide physiological validation of these frameworks in peach.

Transpiration (E) followed the same pattern as photosynthesis (A), with consistently higher rates in 2D trees (*p* < 0.001). Across years and cultivars, E ranged 2.9–6.1 mmol H_2_O m^−2^ s^−1^ in 2D vs. 1.8–5.3 in Quad-V. In ‘Platibelle’, leaves in 2D zones reached 4.1–6.1 compared with 2.5–5.3 in Quad-V, while in ‘Mirel’, values were 2.9–6.1 vs. 1.8–3.8 (Figure 3a). Higher E in 2D reflects the same structural drivers that enhance A, namely, greater radiation reaching interior leaves. Comparable findings in peach show that narrow architectures increase both assimilation and transpiration while maintaining stable water-use efficiency [19,30,43], reinforcing that canopy form strongly regulates gas exchange fluxes.

Interestingly, intercellular CO_2_ concentration (C_i_) was generally statistically significant higher (*p* < 0.05–0.001), mainly at harvest in Quad-V than in 2D. Ranges across years were 121–377 μmol mol^−1^ in 2D vs. 283–383 in Quad-V, with consistent patterns in both cultivars (Figure 3a), suggesting that while 2D systems increased assimilation rates, this occurred at slightly lower internal CO_2_ availability, indicating possibly more efficient CO_2_ drawdown. The lower C_i_ in 2D, combined with higher A, indicates more effective CO_2_ drawdown and tighter stomatal–photosynthetic coupling, consistent with evidence that leaf distribution and microclimate inside canopies modulate leaf functional traits, including stomatal control and photosynthesis [44,45].

Stomatal conductance (g_s_) was significant higher in the 2D system and values ranged 0.14–0.30 mol H_2_O m^−2^ s^−1^ compared with 0.10–0.25 in Quad-V. In ‘Mirel’, the training system effect was more robust and consistent than ‘Platibelle’, while across both years, g_s_ was significantly higher in the 2D system compared to Quad-V in all canopy zones (*p* < 0.001). Enhanced g_s_ in 2D reflects better microclimatic conditions and greater illumination of leaves, favoring stomatal opening likely due to improved light conditions and potentially reduced water stress from more efficient canopy architecture [19,30].

Regarding leaf-to-air vapor pressure deficit (VPD_l_), patterns contrasted between cultivars. In ‘Platibelle’ (2023), VPD_l_ was lower in 2D (*p* < 0.001), while in ‘Mirel’, differences in favor of 2D occurred only at harvest (*p* < 0.001). In 2024, both cultivars showed consistently higher VPD_l_ in 2D, with values ranging 1.6–4.9 kPa compared to 1.5–2.6 in Quad-V (Figure 3b). These contrasting responses suggest that canopy architecture modifies microclimate differently across genotypes. Narrow 2D fruit walls may expose leaves to greater evaporative demand as trees mature, a phenomenon consistent with reports that training alters canopy–atmosphere coupling and the balance between radiation and VPD_l_ as drivers of transpiration [30,46]. Under water deficit, increases in VPD_l_ are known to strongly reduce assimilation, stomatal conductance, and efficiency, marking the onset of negative carbon balance [43]. Recent advances also demonstrate that canopy temperature and related crop water stress indices, strongly influenced by VPD_l_, reliably reflect orchard water status and microclimate differences across training systems [47,48,49].

Water-use efficiency (WUE) showed cultivar-dependent responses. In ‘Platibelle’ (2023), fruiting wall exhibited higher WUE values than Quad-V at both pit hardening and harvest (1.41–1.83 vs. 0.83–1.22 μmol CO_2_ mmol H_2_O^−1^, *p* < 0.01). In ‘Mirel’, Quad-V values were higher at pit hardening in both years (*p* < 0.05), but by 2024 harvest, 2D reached significantly higher WUE (*p* < 0.05–0.001) (Figure 3b). Fruiting walls can enhance WUE under certain developmental and environmental conditions, though cultivar-specific dynamics remain important [30]. Broader evidence indicates that WUE in peach is highly responsive to canopy structure and irrigation strategy: deficit irrigation reduces WUE when water potential falls below critical thresholds [43], while training and pruning interventions can shift canopy balance toward improved efficiency [50,51]. Moreover, canopy temperature-based stress indicators, closely tied to VPD_l_, provide predictive links between gas exchange and WUE dynamics under variable irrigation [47]. These results reinforce that WUE is not a fixed trait but an emergent property of genotype, canopy architecture, and water status, with planar narrow systems offering opportunities for improvement under favorable environmental conditions [52].

Overall, the 2D system substantially improved physiological performance, enhancing A, E, and g_s_, while C_i_ was consistently lower, indicating more efficient CO_2_ drawdown. VPD_l_ and WUE varied with cultivar and year, but in 2024, 2D achieved higher VPD_l_, at both cultivars. These results indicate a more efficient photosynthetic apparatus under fruiting walls, consistent with studies linking improved light distribution to enhanced gas exchange and water-use efficiency in peach and apple [19,30]. Once 50–60% of incident light is intercepted, additional gains become non-linear, making uniform within canopy light penetration the limiting factor for carbon assimilation and fruiting efficiency [22,42]. By reducing self-shading and optimizing light capture during non-noon hours, planar systems enhance stomatal–photosynthetic coupling and can increase leaf-level WUE by sustaining assimilation at lower C_i_ [24,25]. Comparable studies in peach further show that modifying canopy structure or the light environment through asymmetric orchards or protective covers enhances gas exchange while limiting photoinhibition [18,44].

#### 2.2.2. Chlorophyll Fluorescence and Photochemical Efficiency

Chlorophyll fluorescence analysis revealed significant training system effects on photosystem II efficiency (Figure 3b). Effective photochemical efficiency (ΦPSII) was higher in the 2D system than in Quad-V. In ‘Platibelle’, values were significantly higher in 2D, particularly in the second year, while in ‘Mirel’, the advantage was more pronounced across both seasons, with 2D leaves maintaining higher ΦPSII (*p* < 0.01–0.001) compared with Quad-V, reflecting enhanced electron transport efficiency under the improved light environment of planar canopies. Higher ΦPSII in 2D is consistent with reports that canopy modifications can reduce the risk of PSII over-excitation and photoinhibition by moderating excess energy loads on exposed leaves while sustaining photochemistry in inner canopy layers [18]. By maintaining a more balanced leaf energy status, fruiting walls supported greater electron transport and a tighter coupling between gas exchange and photochemistry, as also observed in peaches subjected to altered architectures or protective microclimates [19,44]. Evidence from regulated deficit irrigation trials further highlights that PSII efficiency is highly sensitive to canopy–atmosphere interactions and water status, with narrow planar canopies sustaining higher performance under moderate stress [43,46].

SPAD values, reflecting chlorophyll content, showed significant differences between training systems (*p* < 0.05–0.01) (Figure 3b). Significant but modest increases in SPAD were observed under the 2D system, combined with clear ΦPSII gains, supporting the view that canopy architecture, not chlorophyll amount, was the main driver of physiological improvement [22]. When considered alongside gas exchange data, the fluorescence results reinforce that 2D fruiting walls enable a more balanced use of absorbed light. In detail, at ‘Mirel’, assimilation rates (A) reached up to 9.8 μmol CO_2_ m^−2^ s^−1^ in 2D compared with 4.5 in Quad-V, despite similar SPAD values. This coincided with higher ΦPSII at harvest in 2D (0.24–0.43 vs. 0.12–0.19 in Quad-V), showing that planar canopies sustained higher electron transport while mitigating stress, consistent with findings in narrow orchard systems [14,40].

### 2.3. Canopy Zone Effects on Physiological Performance

In ‘Platibelle’, 2D training system consistently maintained higher overall assimilation, with the difference between upper and lower leaves up to 32.5%. By contrast, in Quad-V the lower canopy frequently showed reduced performance, with signs of stomatal and photosynthetic limitation, while upper leaves only modestly outperformed the shaded strata (Figure 1 and Figure 3a). The 2D system therefore maintained relatively high physiological activity even in lower zones, supporting overall tree performance and more uniform fruit development [1,19,39]. In ‘Mirel’, the contrast between systems was more pronounced. In Quad-V, upper leaves maintained about 10–15% higher photosynthesis than lower ones, indicating persistent vertical heterogeneity. In 2D, however, this stratification was less consistent: in some cases, the upper canopy exceeded the lower by up to one-third, but at harvest, lower leaves often matched or even surpassed the upper, while stomatal conductance and transpiration were generally higher in 2D across both canopy zones (Figure 3a).

These findings reinforce the view that modern training systems should prioritize light availability over maximum interception [24]. Numerous studies in pome and stone fruits confirm that while deep, high-interception canopies often over-shade interior leaves, planar systems sustain more photosynthesis and greater tree-level efficiency [14,22].

### 2.4. Fruit Quality Responses to Training Systems

#### 2.4.1. Physical Traits: Fresh Weight and Firmness

Architecture × crop load x cultivar interactions determine fruit size outcomes; narrow planar systems often trade a slight reduction in per-fruit size for superior uniformity and quality, but cultivar physiology can invert that balance [12,53].

Training system effects on fruit quality were cultivar-dependent, although the direction differed between cultivars (Table 1). In ‘Platibelle’, Quad-V consistently produced heavier fruits than 2D. Ιn ‘Mirel’, the 2D system produced mainly larger fruits, often exceeding Quad-V by 20–25% (2024). These cultivar-specific responses suggest that canopy architecture interacts with genotype in shaping sink strength and fruit growth, consistent with previous reports that light environment and carbon allocation are major drivers of peach fruit size [24,54,55]. Where architectural differences were not significant, higher leaf-to-fruit ratios tended to increase fruit weight by 5–6% g in both systems, confirming assimilate availability as the main driver under load stress [54,55].

Firmness was also training system-dependent, in both cultivars, where 2D fruits were significant firmer than Quad-V, suggesting that planar canopies may favor slower softening and improved textural quality, possibly due to enhanced canopy microclimate and reduced fruit-to-fruit shading [2]. In ‘Mirel’, when no architectural effect was detected, differences were primarily explained by crop load, with higher leaf-to-fruit ratios associated with slightly reduced firmness, especially in Quad-V, aligning with evidence that thinning intensity can modulate firmness through effects on growth rate and cell wall metabolism [14].

Enhanced firmness in fruits has been previously documented and is attributed to improved cell wall development and reduced ethylene production under optimal light conditions [8]. The robust firmness advantage in 2D is consistent with reports that improved within-canopy light elevates dry matter allocation to cell wall components and moderates ethylene-driven softening, enhancing texture at harvest [12].

Collectively, these results indicate that 2D fruiting walls enhance mechanical properties (firmness) in both cultivars, while effects on fresh weight depend on cultivar. ‘Mirel’ generally produced larger fruits, while ‘Platibelle’ maintained superior firmness, reflecting intrinsic genotypic tendencies (Table 1).

#### 2.4.2. Peach Fruit Color Development

Color attributes responded variably to training system, crop load, and cultivar (Table 1).

For lightness (L*), training system effects were significant but cultivar dependent. In ‘Platibelle’, peel lightness decreased under the 2D system only in specific load–zone combinations (*p* < 0.05), indicating darker fruit compared with Quad-V. In contrast, ‘Mirel’ fruit were generally brighter under 2D (e.g., 71.3 vs. 69.8 in Quad-V, *p* < 0.01), highlighting that the influence of canopy structure on brightness is modulated by cultivar characteristics [54].

For red pigmentation (a*), stronger system effects were observed in ‘Platibelle’. Fruits in 2D showed lower a* values than in Quad-V (e.g., 19.9 vs. 13.9; *p* < 0.05), indicating slightly reduced red coloration. In ‘Mirel’, differences between training systems were negligible, as expected for a clingstone cultivar with inherently weaker red pigmentation.

Differences in b* were minor and generally nonsignificant in both cultivars. At the 50 leaves/fruit ratio, ‘Mirel’ fruits often displayed higher b* under 2D (e.g., 60.4 vs. 48.8 in Quad-V; *p* < 0.01), suggesting that crop load exerted a stronger influence than canopy structure on yellow intensity.

For chroma (C*), system effects were weak, while crop load had a clearer impact. In ‘Platibelle’, saturation varied marginally between systems, with significant differences appearing sporadically. In ‘Mirel’, however, fruits under lighter crop loads tended to show higher C* in 2D (e.g., 60.5 vs. 48.9 in Quad-V; *p* < 0.01), indicating enhanced color saturation under improved light conditions when crop competition was reduced.

Hue angle (*h*°) in ‘Platibelle’ tended to be higher under 2D (e.g., 49.0 vs. 57.1; *p* < 0.05), suggesting delayed or less intense red development. ‘Mirel’ showed stable *h*° across systems and crop loads, reflecting strong genetic control over *h*° stability.

Overall, training system influenced specific aspects of peel coloration, particularly lightness in ‘Mirel’ and *h*° in ‘Platibelle’, while crop load and cultivar identity remained dominant determinants of pigment intensity and expression. These findings align with previous reports emphasizing the combined influence of canopy light environment, source–sink balance, and genetic background on peach peel color development [8,9,54,56].

#### 2.4.3. Fruit Quality Parameters

Soluble solids concentration (SSC) exhibited limited responses to training system (Table 1). In ‘Mirel’, a single significant difference emerged at the 50-leaf level (9.6 °Brix in 2D vs. 8.5 in Quad-V; *p* < 0.01), suggesting that SSC was more strongly influenced by crop load than by canopy architecture, particularly under low thinning intensity [54]. This aligns with maturity-controlled evidence that light-rich positions can elevate SSC, though responses are highly cultivar- and vigor-dependent and not universal across training forms [1].

Titratable acidity (TA) displayed clearer cultivar-specific trends. In ‘Mirel’, TA was consistently lower under 2D than in Quad-V (*p* < 0.01). By contrast, ‘Platibelle’ showed no consistent system effect, with differences appearing sporadically between crop loads (e.g., lower TA in 2D at 50 leaves/fruit) but without a stable canopy signal. These patterns indicate that acid metabolism in ‘Mirel’ was more responsive to canopy architecture than in ‘Platibelle’ (Table 1).

Modest SSC shifts but clearer TA reductions, especially in ‘Mirel’, are consistent with studies showing that light environment more reliably influences acid metabolism and flavor balance than bulk sugar accumulation, which is often constrained by crop load and seasonal conditions [12,54].

Dry matter (DM, %) exhibited more frequent training system effects, though again with cultivar dependence (Table 1). In ‘Platibelle’, DM was significantly higher under 2D (*p* < 0.05), while in ‘Mirel’, differences were minor. Enhanced DM under 2D reflects improved light penetration and distribution, which promote assimilate accumulation even when SSC remains unchanged [9]. This relationship has been widely documented in peach, where increased irradiance in interior canopy zones supports greater DM deposition and storability without necessarily increasing SSC uniformly [42,54].

Respiration rate and ethylene production showed minimal sensitivity to training system and only weak modulation by crop load (additional data are given in Table S1). In both cultivars, respiration tended to be slightly higher in Quad-V canopies compared with the planar Four-Axis, but the effect was not systematic across crop load levels.

Overall, these chemical quality responses highlight that both canopy architecture and crop load regulate fruit composition and flavor balance at the tree level. However, mean values may mask substantial within-canopy heterogeneity, particularly in 3D systems with strong vertical light gradients. Consequently, examining canopy zone effects (Section 3.5) provides critical context, revealing how architectural and physiological mechanisms that shape photosynthesis and water relations also govern the spatial distribution of fruit quality [22,56].

### 2.5. Correlation Analysis

**Figure 4:**
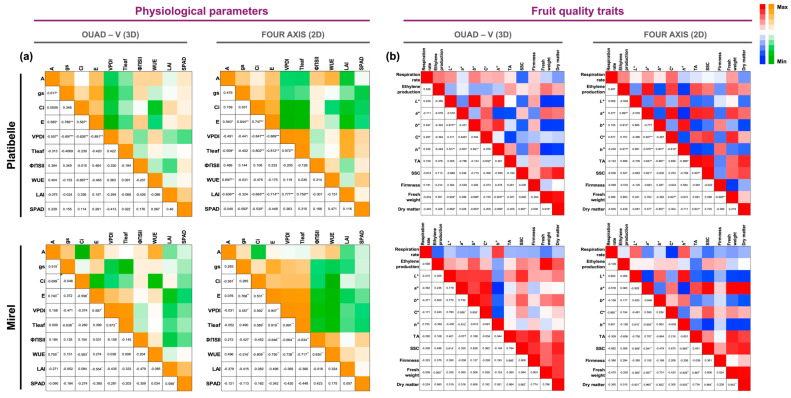
Pearson correlation matrices between (**a**) physiological, canopy structure, and (**b**) fruit quality traits in *Prunus persica* cvs. ‘Platibelle’ and ‘Mirel’ trained under two canopy training systems (Quad-V 3D and Four-Axis 2D). Data represent the mean of the 2023 and 2024 seasons; for fruit quality variables, values also represent the average across thinning levels. The matrices display Pearson correlation coefficients (r) between pairs of variables, with color shading indicating the strength and direction of the relationship (warm tones = positive correlations, cool tones = negative correlations). Significance levels are marked as * (*p* ≤ 0.05) and ** (*p* ≤ 0.01). Variables include A, g_s_, E, C_i_, VPD_l_, WUE, ΦPSII, Tleaf, LAI, SPAD, respiration, ethylene production, fruit weight, firmness, color indices (L*, a*, b*, Chroma; C*, hue angle; *h*°), SSC, TA, and dry matter content.

Correlation analysis revealed strong and coherent relationships among physiological traits across both training systems (Figure 4a).

In ‘Platibelle’ g_s_–SPAD relationship, which was weakly positive in Quad-V (r = 0.16), became moderately negative in Four-Axis (r = −0.59, *p* < 0.05), indicating a reversal in the link between stomatal conductance and leaf chlorophyll status, while VPD_l_–Tleaf rose sharply from moderate in Quad-V (r = 0.42) to high positive in Four-Axis (r = 0.97, *p* < 0.05) and the VPD_l_–LAI correlation switched from weakly negative in Quad-V (r = −0.29) to strongly positive in Four-Axis (r = 0.78, *p* < 0.05), suggesting that greater foliage density increased the effective evaporative surface rather than shading, reinforcing microclimatic feedbacks. Together, these patterns reflect a shift toward stronger thermal and evaporative integration in the planar architecture compared with the more heterogeneous Quad-V system. Within each training system, specific significant associations were also evident. In Quad-V, A correlated positively with g_s_ and E, and negatively with VPD_l_, indicating that g_s_ and E were the main determinants of photosynthetic performance. In contrast, in Four-Axis, photosynthesis showed significant associations with WUE (positive), LAI (negative), and Tleaf (negative), suggesting that carbon assimilation in the planar canopy is more strongly constrained by canopy structure and thermal environment. Moreover, in Four-Axis, C_i_, E, VPD_l_, and LAI formed a tightly coupled set of relationships, reflecting enhanced microclimatic regulation.

Overall, these findings suggest that Quad-V emphasizes direct stomatal and transpiration control of photosynthesis, whereas Four-Axis integrates broader canopy-level factors such as leaf area and temperature, leading to distinct physiological coordination patterns.

In ‘Mirel’, the correlation between A and E decreased sharply from strongly positive in Quad-V (r = 0.74, *p* < 0.01) to negligible in Four-Axis (r = 0.08, ns), while the association between A and WUE also weakened (from r = 0.76 to r = 0.50), indicating that under the planar canopy, carbon assimilation becomes less directly linked to stomatal and transpirational dynamics. Conversely, the relationship between Tleaf and VPD_l_ strengthened markedly (r = 0.97 → 0.99, *p* < 0.05), reflecting tighter microclimatic coupling within the 2D structure. Within each system, distinct internal coordination patterns were evident. In Quad-V, A was strongly and positively correlated with g_s_ and E, and negatively with C_i_, indicating stomatal control of carbon assimilation. The link between E and VPD_l_ confirmed that transpiration was the main driver of leaf gas exchange. In Four-Axis, A correlated weakly with most gas exchange parameters but remained positively related to WUE, while ΦPSII and WUE were jointly constrained by VPD_l_ and Tleaf. This configuration reflects a microclimate-driven photosynthetic regulation, where temperature and vapor conditions dominate over stomatal dynamics.

Overall, these results indicate that canopy architecture substantially restructures the internal physiological coordination of Mirel. The Quad-V system promotes a stomatal/transpirational mode of control, whereas the Four-Axis canopy enhances microclimatic and thermal coupling, leading to a tighter integration between water status, heat load, and photochemical efficiency.

The analysis of fruit quality traits (Figure 4b) in ‘Platibelle’ revealed a remarkable difference in the association between SSC and TA. While in Quad-V this relationship was absent (r = −0.16), under Four-Axis it became strongly positive (r = 0.91, *p* < 0.05), suggesting that in the planar canopy system, sugar accumulation and acid retention are more tightly coupled, potentially leading to a more balanced flavor profile.

Beyond this statistically significant difference, broader comparison of correlation patterns indicated distinct system-specific trends. In Quad-V, fruit fresh weight and dry matter were tightly linked, implying that fruit size was a primary determinant of compositional quality. In contrast, under the Four-Axis system, dry matter was more strongly associated with TA and SSC, highlighting a shift toward a quality-driven metabolic integration rather than size dependence. Color attributes also reflected differences in canopy architecture. Quad-V displayed strong associations between L*, *h*°, and the b* coordinate, whereas the Four-Axis system showed extremely strong negative correlations among a*, C*, and *h*°, indicating a more uniform coupling of chromatic traits. Importantly, SSC was more closely integrated with TA and chroma in the Four-Axis canopy, while in Quad-V it related primarily to dry matter.

The comparison of fruit quality correlations between training systems in ‘Mirel’ revealed a profound reorganization of color, compositional, and respiratory relationships. In the Four-Axis system, the correlation between respiration rate and chroma shifted from weakly negative in Quad-V (r = −0.17) to extremely strong and negative (r = −0.97, *p* < 0.01), indicating that enhanced respiratory activity was closely linked to pigment degradation and loss of color saturation. Likewise, the *L*–a* relationship inverted from positive (r = 0.78) to strongly negative (r = −0.93, *p* < 0.01), suggesting that in the planar canopy, brighter fruits tended to be less red.

Color coordination also became more tightly integrated in the 2D system: the correlations among a*, C*, and *h*° were all stronger and more negative (r = −0.99 to −0.93), revealing a more rigid coupling of chromatic parameters. Simultaneously, the association between SSC and *h*° became markedly stronger (r = −0.97, *p* < 0.01), reflecting a closer alignment between sugar accumulation and color development under the planar canopy. Conversely, the SSC—TA relationship weakened (r = 0.79 → 0.45), indicating a partial decoupling of sugar and acid accumulation.

Within each system, distinct structural patterns emerged. In Quad-V, fruit color variables (L*, a*, b*, *h*°) were moderately intercorrelated, and quality was primarily size-driven, as shown by the positive association between fresh weight and dry matter (r = 0.77, *p* < 0.05). Under Four-Axis, color, SSC, and TA formed a tightly integrated cluster, whereas size-related traits remained correlated but subordinate. This pattern suggests that the planar architecture enhances the metabolic coordination of ripening processes, linking pigment synthesis, sugar concentration, and respiratory dynamics, while the open vase system maintains a looser, size-oriented structure.

Across cultivars, the planar Four-Axis system consistently enhanced thermal, gas exchange, and compositional coupling, while Quad-V preserved more independent, size-based relationships. ‘Platibelle’ displayed stronger stomatal and structural contrasts, whereas ‘Mirel’ exhibited deeper metabolic and chromatic reorganization. Collectively, these results demonstrate that canopy architecture and cultivar physiology jointly shape the intensity and direction of functional trait linkages, shifting coordination from morphology-driven in Quad-V to metabolically integrated in Four-Axis systems. These results are consistent with previous findings in simplified architectures [18]. Across crop load levels, fruit traits segregated into two clusters: (i) structural/compositional traits (firmness, SSC, DM, fresh weight) and (ii) color attributes (a*, C*, *h*°). Significant negative correlations between *h*° and both a* and C* consistently indicated that improved red pigmentation was tightly coupled with higher saturation. The greater number and strength of significant correlations at the 50-leaf ratio suggest that reduced crop load promotes stronger trait integration (data are given in Figure S2).

### 2.6. Impact of Training Systems on Yield Efficiency and Total Production

**Figure 5:**
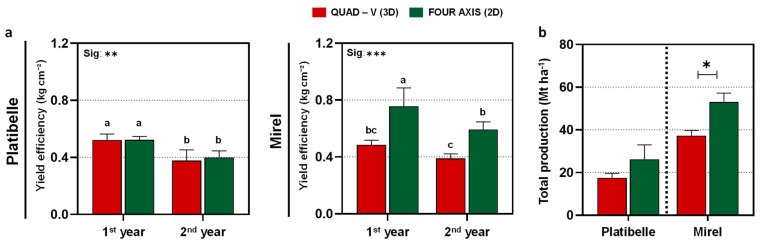
Yield efficiency and orchard productivity of *Prunus persica* cvs. ‘Platibelle’ and ‘Mirel’ in two canopy architectures, Quad-V (3D, red) and Four-Axis (2D, green), across two consecutive growing seasons (2023–2024). (**a**) Yield efficiency (kg cm^−2^ trunk cross-sectional area, TCSA) and (**b**) total orchard production (Mt ha^−1^, calculated from tree yield and planting density) were assessed to evaluate the cumulative impact of training system and cultivar on orchard productivity. Bars represent mean values ± SE (n = 4). Different letters indicate statistically significant differences among treatments according to Duncan’s multiple range test (*p* ≤ 0.05). Significance between training systems is denoted as * *p* ≤ 0.05, ** *p* ≤ 0.01, and *** *p* ≤ 0.001 (ANOVA); *t*-test results comparing mean productivity between training systems are also reported where relevant.

Yield efficiency showed cultivar- and training system-specific responses (Figure 5a). In ‘Platibelle’, no consistent training system differences were detected across years, with the average yield efficiency remaining stable at 0.45 kg cm^−2^, with no significant separation between systems. In contrast, ‘Mirel’ exhibited strong training system effects (*p* < 0.001). Τhe 2D maintained consistently higher efficiency (0.68 vs. 0.44 kg cm^−2^ in Quad-V), confirming the advantage of planar canopies in supporting higher productivity per unit trunk cross-sectional area [2,9,20].

Patterns for total production (Mt ha^−1^) further underlined these differences (Figure 5b). In ‘Platibelle’, yields were numerically higher in 2D (26.25 t ha^−1^) compared with Quad-V (17.50 t ha^−1^), but these contrasts were not statistically significant given the higher within-system variation. In ‘Mirel’, the 2D system produced consistently greater yields (53.0 t ha^−1^) than Quad-V (37.3 t ha^−1^), with a statistically significant advantage (*p* < 0.05). Gains of 42% over the two-year average highlight the strong alignment between ‘Mirel’s’ growth habit, reflecting the combined effects of higher efficiency and improved light availability in 2D systems [12].

Overall, these results indicate that training system effects on yield efficiency and total production are cultivar-dependent. In ‘Platibelle’, total production was variable higher but not significant under 2D, reaching up to a 50% increase compared to Quad-V, whereas in ‘Mirel’, the advantage of 2D was consistently strong across both efficiency and yield. This divergence highlights the importance of aligning canopy architecture with cultivar-specific growth and fruiting patterns to maximize orchard performance. Broader evidence confirms that narrow planar systems not only support higher productivity but also improve pack out rates, fruit uniformity, and labor efficiency, while providing orchard designs compatible with future mechanization and automation [14,26]. More generally, orchard design that balances light interception with its effective distribution maximizes usable photosynthesis and economic returns, rather than maximizing interception per tree [22,42].

### 2.7. Impact of LAI on Total Production and Fresh Weight

**Figure 6:**
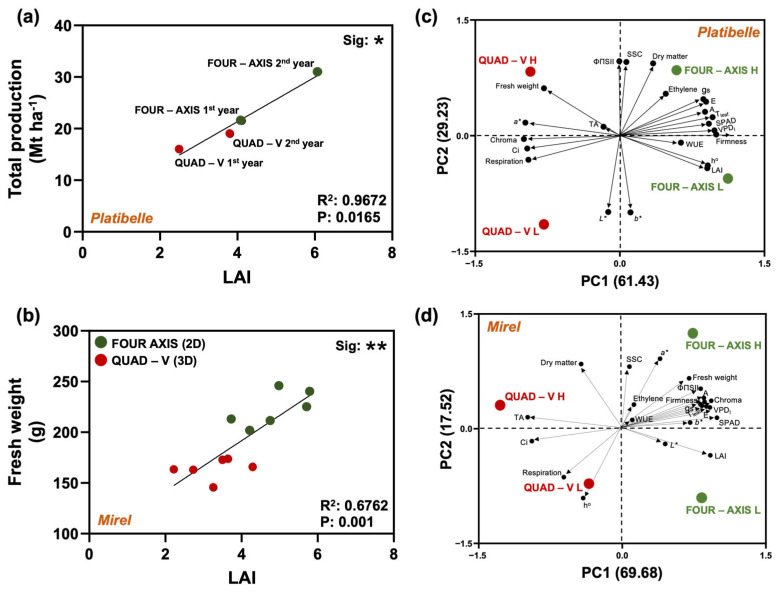
Multivariate relationships between canopy vigor, physiological performance, and fruit quality in *Prunus persica* cvs. ‘Platibelle’ and ‘Mirel’ trained to two canopy architectures (Quad-V 3D and Four-Axis 2D). Panel (**a**) depicts the regression between canopy development (LAI) and total orchard productivity (Mt ha^−1^) across training systems in ‘Platibelle’ and (**b**) across fresh weight in ‘Mirel’ for two consecutive years. Principal component analyses (PCA) integrate physiological (A, g_s_, E, C_i_, ΦPSII, SPAD, Tleaf, WUE, LAI, VPD_l_), and fruit quality attributes (fresh weight, firmness, color indices, SSC, TA, dry matter), together with ethylene production and respiration rate for two consecutive years (2023 and 2024) and two thinning levels (20 and 50 leaves), to visualize clustering patterns across canopy zones (L = lower, H = upper) in (**c**) Platibelle and (**d**) Mirel. Large filled circles represent the training systems and canopy zones (green = Four-Axis 2D, red = Quad-V 3D), whereas smaller circles denote the variable loadings. The PCA biplots highlight distinct separation of training systems along PC1, mainly driven by variation in leaf physiological activity and photosynthetic performance. In both cultivars, Four-Axis system clustered toward positive PC1 scores, associated with higher canopy vigor and photosynthetic activity, while Quad-V grouped toward negative PC1 values, corresponding to lower light availability but enhanced compositional traits. Significance between training systems is denoted as * *p* ≤ 0.05, ** *p* ≤ 0.01. Extraction method: Principal Component Analysis; rotation method: Varimax with Kaiser normalization.

Across training systems, total production generally increased with LAI, though with diminishing returns and clear system- and cultivar-specific optima. In ‘Platibelle’, a strong positive linear relationship was found between LAI and yield (R^2^ = 0.97, *p* = 0.0165) (Figure 6a). Increasing LAI within the 2D system from 4.1 to 6.0 was associated with a 44% yield gain (21.5 → 31.0 t ha^−1^), whereas the same increase in the 3D canopy (2.5 → 3.8) produced only a 19% gain (16.0 → 19.0 t ha^−1^), indicating that planar architectures convert additional leaf area into fruit more efficiently at moderate LAI.

In ‘Mirel’, LAI was positively correlated with mean fruit fresh weight (R^2^ = 0.68, *p* = 0.001) (Figure 6b), but 2D canopies showed signs of over-foliation: increasing LAI from 3.3 to 5.0 slightly reduced yield (56 → 50 t ha^−1^) (additional data are given in Figure S3), suggesting an optimal LAI band below 5.0 for this cultivar in planar systems.

Light availability was positively associated with dry matter accumulation in ‘Platibelle’, while fresh weight in ‘Mirel’, indicating stronger light-use efficiency for biomass formation (Figure S3).

These trends support a concave (saturating) LAI–yield relationship, where yield initially rises with increasing leaf area due to enhanced light interception, but plateaus or declines beyond a threshold as self-shading, vigor-related costs, and canopy atmosphere decoupling accumulate.

This behavior aligns with long-established tree-crop theory: (i) yield scales with LAI or LI up to a critical point, after which excess foliage adds interception without proportional gains in canopy photosynthesis or fruit growth due to saturation, photoinhibition, or vigor diversion [18,24], (ii) orchard-level studies confirm this relationship, noting large variability at high-interception levels because excess vigor or poor internal illumination reduces efficiency [14,42], and (iii) planar systems more effectively utilize moderate LAI by maintaining photosynthetically active light in fruiting zones, whereas excessive LAI, even in 2D canopies, induces shading, respiratory losses, and vigor penalties [2,9].

Water status can further modulate this response under stress, high-light leaves lose photosynthetic efficiency more rapidly than shaded leaves, flattening the LAI–yield curve [14].

Overall, 2D canopies achieved higher yields at moderate LAI (e.g., ‘Platibelle’), but overshooting optimal foliage levels (e.g., ‘Mirel’) reduced production. Thus, managing LAI within cultivar-specific optima, rather than maximizing canopy density, is critical to optimizing usable light, canopy photosynthesis, and yield in modern peach orchards (additional data are given in Figure S3).

### 2.8. PCA Analysis

The PCA analysis further highlighted the contrasting coordination of traits under different training systems and canopy zones, summarizing most of the total variance in the first two components (‘Platibelle’: PC1 = 61.4%, PC2 = 29.2%; ‘Mirel’: PC1 = 69.7%, PC2 = 17.5%) (Figure 6c,d). In both cultivars, PC1 primarily distinguished training systems, whereas PC2 primarily separated the canopy zones. Quad-V and Four-Axis configurations formed partially distinct clusters along PC1 (Quad–V was positioned toward the negative side of PC1, whereas the Four-Axis system positive), confirming that training system architecture reorganizes the multivariate structure of plant performance.

In ‘Platibelle’, PC1 was dominated by gas exchange/thermal and leaf-status variables with high positive loadings: A (0.884), g_s_ (0.866), E (0.895), VPD_l_ (0.981), Tleaf (0.962), LAI (0.907), SPAD (0.923). Among color traits, *h*° loaded positively on PC1 (0.912), while a* and C* loaded negatively (−0.980 and −0.995). PC2 carried SSC (0.956) and DM (0.938) positively, together with ΦPSII (0.966), and had strong negative loadings for L* (−0.989) and b* (−0.994). PC3 concentrated WUE (0.768), ethylene production (0.691), and TA (0.979) (Figure 6c). In ‘Mirel’, PC1 again showed high positive loadings for gas exchange/thermal and leaf-status variables: A (0.852), g_s_ (0.832), E (0.880), VPD_l_ (0.916), Tleaf (0.930), LAI (0.920), SPAD (0.989), with C_i_ (−0.945) and respiration rate (−0.611) negative. Unlike ‘Platibelle’, color metrics were mainly positive on PC1 (C* 0.859, b* 0.709, L* 0.449) and TA loaded strongly negative (−0.988). PC2 captured compositional and color separation with positive loadings for SSC (0.812), DM (0.845), a* (0.915) and a strong negative loading for *h*° (−0.907) (Figure 6d).

Across cultivars, PC1 represents a physiological/microclimatic axis that separates the training systems; PC2 encodes the upper–lower canopy contrast and is associated with compositional and color attributes, with cultivar-specific patterns in color loadings (negative a*/C* in ‘Platibelle’ vs. positive a*/C* in ‘Mirel’). Together, the PCA underscores that training system and canopy zone jointly determine the physiological–quality trait networks.

## 3. Materials and Methods

### 3.1. Experimental Design and Plant Material

The experiment was carried out during the 2023 and 2024 growing seasons, from full bloom (early March) until commercial harvest (early July for ‘Platibelle’ and late July for ‘Mirel’), in commercial orchards at Imathia, Central Macedonia, Greece (40°34′52.10″ N, 22°20′57.35″ E), the country’s leading peach production region [36]. The Mediterranean climate features warm, dry summers (mean summer temperature 28 °C) and mild winters, with annual precipitation around 600 mm. Two peach cultivars were evaluated: 5-year-old ‘Platibelle,’ an early-maturing flat peach for the fresh market, and 6-year-old ‘Mirel,’ a mid-season clingstone processing cultivar. Both cultivars were grafted onto GF-677 rootstock to ensure vigor and adaptability to calcareous soils. All orchard rows were oriented north–south to optimize diurnal light interception [9]. Physiological measurements were performed at pit hardening and commercial maturity, while fruit quality traits were conducted at commercial harvest.

### 3.2. Training Systems and Experimental Design

The experiment followed a completely randomized design comparing two training systems. In the two-dimensional (2D) planar fruiting wall system, trees were spaced 3.5 m between rows and 2.8 m within rows (1020 trees ha^−1^) and trained to a planar canopy with four vertical axes. In the three-dimensional (3D) Quad-V system, trees were arranged with 4.7 m inter-row and 3.6 m intra-row spacing (590 trees ha^−1^) and trained to a V-shaped canopy with four scaffolds angled at 45° from vertical [28]. The selected spacing dimensions reflect practical commercial constraints and canopy uniformity requirements rather than a theoretical universal optimum. Eight trees per cultivar and training system (32 trees in total) were selected for uniform vigor [9]. All trees were managed under integrated pest management practices, with identical fertilization, irrigation, and pest-control regimens across treatments. Each tree canopy was divided into two zones according to height and light exposure: the lower canopy (0–1.2 m above ground) and the upper canopy (1.8–3.3 m). Within each zone, two representative shoots (east- and west-facing) were tagged at approximately 1.0 m in the lower zone and 2.2 m in the upper zone. Two fully expanded leaves per tagged shoot were selected for physiological measurements [1].

To evaluate the interaction between source–sink balance and fruit quality, two thinning regimes were applied within each canopy zone: a “low crop load” (50 leaves per fruit) and a “high crop load” (20 leaves per fruit). These specific leaf-to-fruit ratios were selected to represent commercially relevant thresholds for sink-limited and source-limited conditions in peach, based on established physiological standards [2,6,7,14,53].

### 3.3. Physiological Measurements

#### 3.3.1. Estimation of Leaf Area Index (LAI)

Leaf area index (LAI) was measured non-destructively during the two growing seasons using a LI-2200 Plant Canopy Analyzer (LI-COR Biosciences, Lincoln, NE, USA). Measurements were taken at four vertical canopy positions: 25 cm above the graft union, and at 100 cm, 180 cm, and 260 cm above the orchard floor. All measurements were made 30 cm from the trunk, and the values were averaged per canopy height zone to assess vertical canopy density distribution [1,25].

#### 3.3.2. Canopy Light Environment

Photosynthetically active radiation (PAR) was measured at multiple positions within each canopy zone using an MQ-301 Line Quantum Sensor with 10 sensors (Apogee Instruments, Logan, UT, USA). Light availability (LA, %) was calculated as the average PAR at the canopy position divided by incident PAR, multiplied by 100, following the methodology of [44].

The light distribution index (LDI) was computed as the difference between upper and lower canopy light availability divided by their sum [9].

$$\mathrm{LDI}=\frac{\left(\mathrm{Light}\;\mathrm{availability}\;\%\;\mathrm{at}\;2.2\;m\right)-\left(\mathrm{Light}\;\mathrm{availability}\;\%\;\mathrm{at}\;0.8\;m\right)}{\left(\mathrm{Light}\;\mathrm{availability}\;\%\;\mathrm{at}\;2.2\;m\right)+\left(\mathrm{Light}\;\mathrm{availability}\;\%\;\mathrm{at}\;0.8\;m\right)}$$

LDI values range from 0 to 1, where values approaching 0 indicate highly heterogeneous light distribution, and values approaching 1 indicate uniform light distribution.

Light interception was calculated using the inverse of light availability:

Light interception (LI) (%) = 100 − Light availability (%)

This approach quantifies the proportion of incoming radiation absorbed by the canopy at different heights and provides insight into structural efficiency and canopy closure.

#### 3.3.3. Leaf Gas Exchange

Leaf gas exchange parameters were measured under clear skies using a LI-6400XT Portable Photosynthesis System (LI-COR Inc., Lincoln, NE, USA). The system maintained CO_2_ concentration at 420 µmol mol^−1^ and ambient humidity. Net photosynthetic rate (A, µmol CO_2_ m^−2^ s^−1^), stomatal conductance (g_s_, mol H_2_O m^−2^ s^−1^), transpiration rate (E, mmol H_2_O m^−2^ s^−1^), intercellular CO_2_ concentration (C_i_, µmol CO_2_ mol^−1^ air), VPD_l_ (kPa) and Tleaf (°C) were recorded [18,57]. Instantaneous water-use efficiency was calculated as A/E (WUE, µmol CO_2_ mmol H_2_O^−1^) [58].

#### 3.3.4. Chlorophyll Fluorescence and Chlorophyll Content

Chlorophyll fluorescence parameters were determined using an OS-5p chlorophyll fluorometer (Opti-Sciences, Tyngsboro, MA, USA) and the effective photochemical efficiency (ΦPSII) was recorded [59]. Leaf chlorophyll concentration was assessed with a SPAD-502 Plus meter (Konica Minolta, Osaka, Japan), taking three readings per leaf [60].

### 3.4. Fruit Quality Analysis

#### 3.4.1. Harvest and Sampling

For fruit quality analysis, 10 fruits were randomly sampled from each of the four replicate trees per treatment combination, separated by canopy zone (upper and lower). Fruits were harvested at commercial maturity based on a combination of maturity indices, including background peel color, flesh firmness [61], and the DA-meter index (difference in absorbance between 670 and 720 nm) [32]. Only visually uniform, defect-free fruits were selected for analysis. Respiration rate and ethylene production were conducted with the Shimadzu GC—2014 (Kyoto, Japan) and the results were expressed as mL CO_2_ kg^−1^ h^−1^ and μL C_2_H_4_ kg^−1^ h^−1^, respectively.

#### 3.4.2. Physical and Color Parameters

Fruit fresh weight was determined with an electronic balance, while flesh firmness was measured with FTA GS-25 Fruit Texture Analyser (UP Umweltanalytische Produkte GmbH, Cottbus, Germany) at two equatorial positions using an 8 mm-probe penetrometer [62]. Fruit dimensions were recorded using digital calipers. Skin color parameters (L*, a*, b*) were obtained with a CR410 Chroma Meter (Konica Minolta, Tokyo, Japan), and chroma (C*) and hue angle (*h*°) were derived mathematically from a* and b* values [63].

#### 3.4.3. Chemical Composition

Soluble solids concentration (SSC, °Brix, %) was measured using a portable electronic refractometer (Atago Co., Ltd., Tokyo, Japan). Titratable acidity (TA, % malic acid) was determined by titration with 0.1 N NaOH with an automatic titrator (HI 84532, Hanna Instruments, Woonsocket, RI, USA). Dry matter content (DM, %) was obtained by oven-drying subsamples.

### 3.5. Evaluation of Tree Yield and Orchard Productivity

Harvest was conducted across two picks for ‘Mirel’ and three picks for ‘Platibelle’, based on the canopy/ripening uniformity of each training system. Yield (kg tree^−1^) and average fruit weight (FW, g) were determined by counting and weighing the harvested fruits. Orchard productivity was expressed as metric tonnes per hectare (MT ha^−1^), calculated based on individual tree yield and planting density specific to each training system (1020 trees ha^−1^ for the 2D system; 590 trees ha^−1^ for the 3D Quad-V system).

Trunk cross-sectional area (TCSA) was measured in November of each season at 15 cm above the graft union using a digital caliper. Yield efficiency (YE, kg cm^−2^) was calculated as the ratio of total yield per tree (kg) to TCSA (cm^2^), following standard protocols used in peach fruit [24]. Yield efficiency was expressed in kilograms of fruit per square centimeter of trunk cross-section (kg cm^−2^).

Yield efficiency (YE) was calculated following established methodology as

$$\mathrm{YE}=\frac{\mathrm{Total}\;\mathrm{yield}\;\mathrm{per}\;\mathrm{tree}\;(\mathrm{kg})}{{\mathrm{TCSA}\;(\mathrm{cm}}^{2})}$$

### 3.6. Statistical Analysis

Data were analyzed separately for each growing season. Data were subjected to two-way ANOVA with training system and canopy zone as main factors for physiology measurements. Means were compared using Duncan’s multiple range test at *p* < 0.05. For fruit quality traits, a multifactor analysis of variance (ANOVA) was performed including training system, canopy zone and thinning level as fixed factors. When significant effects were detected, mean separation was performed using Duncan’s multiple range test (*p* ≤ 0.05). In addition, pairwise *t*-tests were used to compare training systems within the same canopy zone and thinning level. Pearson correlation coefficients evaluated relationships among physiological, canopy, and fruit quality parameters. Principal Component Analysis (PCA) was conducted to visualize group separation between 2D fruiting walls and 3D Quad-V groups and canopy zones as well as to identify the main physiological, light-related, and fruit quality variables contributing to sample variance. All statistical analyses were performed with SPSS v25.0 (IBM Corp., Armonk, NY, USA), and figures were prepared using GraphPad Prism v10.2.0 (GraphPad Inc., San Diego, CA, USA).

## 4. Conclusions

Canopy architecture emerged as a primary driver of the light environment, physiological performance, and fruit quality in peach. Compared to 3D Quad-V systems, planar 2D canopies carried higher LAI and LI. Although Quad-V intercepted more total light, it also intensified vertical stratification, leading to less favorable within-canopy conditions. In contrast, leaf gas exchange parameters (A, E, g_s_) were consistently enhanced in 2D canopies, while lower C_i_ values indicated more effective CO_2_ drawdown under improved light penetration. The higher ΦPSII observed in 2D supports greater electron transport efficiency under a moderated energy load. Finally, WUE responses varied with cultivar and microclimate but generally followed the shifts observed in VPD_l_ and Tleaf, reinforcing the central role of canopy design in shaping whole-plant function and productivity.

Canopy zone contrasts were significantly modulated by the training system. While vertical gradients were not eliminated, the 2D system mitigated the physiological penalty typically associated with lower canopy positions. Specifically, fruiting walls sustained photosynthetic activity in the lower strata at levels significantly higher than the 3D Quad-V system, in some cases comparable to the upper canopy. Cultivar-specific differences further highlighted the interaction between architecture and genotype. In ‘Platibelle’, 2D canopies improved firmness and dry matter without penalizing yield, whereas ‘Mirel’ responded with greater fruit size and significantly higher productivity per unit trunk area.

At the orchard scale, yield and light-use efficiency followed a saturating response to LAI, with 2D canopies converting moderate foliage levels into fruit biomass more effectively. Over-foliation, however, reduced yield, underscoring the need for cultivar-specific LAI optimization. Summarizing, these findings indicate that optimizing canopy structure enhances photosynthetic efficiency, fruit quality, and yield sustainability; 2D planar canopies thus represent a viable framework for high-density, resource-efficient, and mechanization-compatible peach production systems.

These conclusions align with the global trend toward intensification in peach production through adoption of high-density planar training systems. While initial establishment costs are higher due to increased tree density, the improvements in fruit quality, production efficiency, and mechanization potential justify the investment, particularly for fresh market production systems. The choice of training system should be tailored not only to orchard management objectives but also to cultivar-specific growth and yield responses. Future research should focus on optimizing cultural practices specific to planar systems, including precision crop load management, irrigation strategies adapted to higher tree densities, and development of mechanization technologies compatible with narrow canopy architectures.

## Figures and Tables

**Table 1:** Effect of training system (Quad-V 3D vs. Four-Axis 2D), canopy position (low vs. high), and thinning level (20 and 50 leaves per fruit) on fruit physicochemical traits of *Prunus persica* cvs. (a) ‘Platibelle’ and (b) ‘Mirel’ during the 2023 and 2024 growing seasons. Data represent mean values ± standard deviation (n = 3). Different letters within rows indicate statistically significant differences between training systems according to Duncan’s multiple range test (*p* ≤ 0.05) within each cropping year. *t*-test results comparing training systems within the same canopy zone are also reported where relevant. Significance levels: * *p* ≤ 0.05; ** *p* ≤ 0.01; *** *p* ≤ 0.001; ns = not significant.

				Platibelle									Mirel								
Quality Traits	Year	Canopy Zone	Thinning Level (Leaves/ Fruit)	Quad-V (3D) (Mean ± SD)				Four-Axis (2D) (Mean ± SD)				*t*-Test (*p*-Value)	Quad-V (3D) (Mean ± SD)				Four-Axis (2D) (Mean ± SD)				*t*-Test (*p*-Value)
Fresh weight (g)	2023	Low	20	117.0	±	16.8	cd	97.2	±	13.1	f	***	164.1	±	14.5	b	164.6	±	18.4	b	ns
			50	125.9	±	24.0	bc	100.4	±	17.9	ef	***	169.3	±	17.5	b	175.2	±	17.2	b	ns
		High	20	139.8	±	19.7	a	111.6	±	12.5	de	***	194.9	±	23.2	a	176.7	±	16.5	b	*
			50	134.0	±	23.7	ab	113.0	±	18.9	cde	**	192.5	±	28.5	a	190.8	±	20.0	a	ns
	2024	Low	20	131.8	±	19.2	cd	118.3	±	15.8	d	*	163.4	±	23.0	d	218.2	±	32.3	bc	***
			50	137.1	±	17.4	c	131.1	±	18.2	cd	ns	173.4	±	19.0	d	204.6	±	29.0	c	***
		High	20	154.1	±	25.6	ab	141.2	±	18.5	bc	ns	160.9	±	37.0	d	240.9	±	24.8	a	***
			50	162.5	±	19.4	a	154.1	±	35.9	ab	ns	163.2	±	18.0	d	226.4	±	40.1	ab	***
Firmness (kg)	2023	Low	20	6.2	±	0.6	c	7.1	±	0.5	a	***	4.6	±	0.7	bc	5.1	±	0.7	a	*
			50	5.4	±	0.6	d	7.2	±	0.6	a	***	4.2	±	0.6	c	4.7	±	0.6	ab	**
		High	20	6.1	±	0.5	c	6.6	±	0.7	b	*	4.3	±	0.6	bc	4.8	±	0.5	ab	*
			50	6.0	±	0.5	c	7.1	±	0.5	a	***	4.4	±	0.8	bc	4.8	±	0.7	ab	ns
	2024	Low	20	4.8	±	0.4	abc	4.9	±	0.5	abc	ns	4.4	±	0.5	ab	4.4	±	0.5	ab	ns
			50	5.0	±	0.7	ab	5.1	±	0.6	a	ns	3.8	±	0.5	c	4.6	±	0.5	a	***
		High	20	4.6	±	0.8	bc	4.8	±	0.5	abc	ns	4.5	±	0.4	a	4.7	±	0.4	a	ns
			50	4.5	±	0.6	c	5.2	±	0.7	a	**	4.2	±	0.5	b	4.6	±	0.4	a	**
*Lightness* (L*)	2023	Low	20	52.4	±	1.8		52.4	±	2.9		ns	69.8	±	0.3	cd	71.3	±	0.2	bc	**
			50	55.2	±	1.1		51.3	±	1.8		*	69.3	±	0.6	d	79.0	±	1.5	a	***
		High	20	49.6	±	1.8		51.0	±	1.1		ns	68.8	±	0.1	d	71.9	±	0.6	b	***
			50	53.3	±	1.5		51.6	±	1.8		ns	69.0	±	1.9	d	69.0	±	0.2	d	ns
	2024	Low	20	40.5	±	1.0	bc	42.9	±	2.3	ab	ns	60.9	±	4.9	c	66.7	±	2.5	ab	ns
			50	47.8	±	2.3	a	42.4	±	2.2	b	*	66.1	±	1.1	ab	62.5	±	2.3	bc	ns
		High	20	36.6	±	2.9	c	38.8	±	2.0	bc	ns	67.9	±	2.0	a	64.6	±	1.5	abc	ns
			50	41.2	±	3.1	bc	39.0	±	5.6	bc	ns	66.6	±	1.6	ab	65.8	±	1.2	ab	ns
*Redness* (a*)	2023	Low	20	19.9	±	2.7	ab	13.9	±	2.3	c	*	-3.8	±	1.1	c	-3.4	±	0.9	c	ns
			50	19.9	±	1.5	ab	15.2	±	1.9	c	*	-2.2	±	1.8	abc	-2.4	±	1.2	bc	ns
		High	20	20.8	±	0.2	a	17.2	±	1.7	abc	*	-0.6	±	1.4	ab	-1.2	±	0.6	ab	ns
			50	19.3	±	2.1	ab	16.8	±	2.9	bc	ns	-0.1	±	0.6	a	-0.5	±	0.4	ab	ns
	2024	Low	20	37.3	±	4.6	a	25.0	±	4.6	b	*	38.1	±	3.4	bc	41.1	±	2.7	bc	ns
			50	37.4	±	3.8	a	27.4	±	1.9	b	*	41.1	±	1.4	bc	38.7	±	4.3	bc	ns
		High	20	38.5	±	0.5	a	35.9	±	4.6	a	ns	40.2	±	1.8	bc	43.2	±	0.9	ab	ns
			50	41.1	±	1.2	a	28.3	±	1.8	b	***	36.6	±	2.8	c	46.9	±	3.0	a	*
*Yellowness* (b*)	2023	Low	20	22.5	±	0.4	ab	22.7	±	1.3	ab	ns	50.1	±	0.6	cd	52.1	±	1.4	c	ns
			50	22.8	±	0.1	ab	23.6	±	0.4	a	*	48.8	±	2.8	d	60.4	±	1.9	a	**
		High	20	20.9	±	1.1	c	22.1	±	0.3	bc	ns	49.7	±	0.7	cd	55.3	±	2.7	b	*
			50	22.0	±	0.4	bc	22.6	±	0.6	ab	ns	50.5	±	0.7	cd	51.0	±	0.8	cd	ns
	2024	Low	20	24.3	±	0.3	bc	24.4	±	0.6	bc	ns	41.5	±	3.4		45.1	±	2.0		ns
			50	28.7	±	1.4	a	26.1	±	2.2	ab	ns	44.9	±	0.6		42.4	±	1.9		ns
		High	20	21.8	±	2.0	c	23.3	±	1.2	bc	ns	45.9	±	1.4		43.7	±	1.8		ns
			50	24.9	±	1.6	bc	22.3	±	3.8	c	ns	44.4	±	1.6		45.5	±	0.5		ns
Chroma (C*)	2023	Low	20	30.1	±	2.0		26.6	±	1.3		ns	50.3	±	0.5	cd	52.2	±	1.4	c	ns
			50	30.3	±	1.0		28.1	±	1.1		ns	48.9	±	2.8	d	60.5	±	1.8	a	**
		High	20	29.5	±	0.7		28.0	±	0.8		ns	49.7	±	0.7	cd	55.3	±	2.7	b	*
			50	29.3	±	1.7		28.2	±	1.7		ns	50.6	±	0.7	cd	51.0	±	0.8	cd	ns
	2024	Low	20	44.6	±	4.0	a	35.0	±	3.6	b	*	56.3	±	4.6	b	61.0	±	3.3	ab	ns
			50	47.2	±	3.3	a	37.9	±	2.5	b	*	60.8	±	1.3	ab	57.4	±	4.3	b	ns
		High	20	44.3	±	1.1	a	42.9	±	3.4	a	ns	61.0	±	0.7	ab	61.4	±	1.1	ab	ns
			50	48.1	±	0.5	a	36.1	±	2.5	b	**	57.5	±	2.8	b	65.4	±	2.5	a	*
Hue angle (*h*°)	2023	Low	20	48.6	±	3.6	bc	58.6	±	5.0	a	*	94.4	±	1.3	a	93.8	±	1.0	a	ns
			50	49.0	±	2.2	bc	57.2	±	3.3	a	*	92.6	±	2.0	ab	92.3	±	1.2	abc	ns
		High	20	45.1	±	1.7	c	52.3	±	3.1	ab	*	90.7	±	1.7	bc	91.2	±	0.6	bc	ns
			50	48.8	±	2.7	bc	53.6	±	5.0	ab	ns	90.2	±	0.6	c	90.6	±	0.4	bc	ns
	2024	Low	20	33.3	±	2.9	cd	44.6	±	4.7	a	*	47.4	±	1.2	bc	47.6	±	0.6	bc	ns
			50	37.6	±	3.0	bc	43.6	±	2.2	ab	*	47.6	±	0.7	bc	47.8	±	2.0	bc	ns
		High	20	29.4	±	2.2	d	33.2	±	4.4	cd	ns	48.7	±	2.0	ab	45.3	±	1.6	cd	ns
			50	31.2	±	2.4	cd	38.0	±	5.4	bc	ns	50.5	±	1.5	a	44.1	±	1.6	d	**
SSC (Brix, %)	2023	Low	20	11.9	±	0.3		11.5	±	0.0		ns	8.7	±	0.2	ab	8.8	±	0.6	ab	ns
			50	11.1	±	1.4		12.3	±	0.4		ns	8.5	±	0.2	b	9.6	±	0.1	a	**
		High	20	11.6	±	1.1		12.5	±	0.7		ns	9.0	±	1.0	ab	9.6	±	0.6	a	ns
			50	12.6	±	0.4		13.0	±	0.1		ns	9.6	±	0.3	a	9.6	±	0.2	a	ns
	2024	Low	20	11.4	±	0.2	c	11.5	±	1.1	c	ns	9.9	±	0.6	e	10.6	±	0.1	bcd	ns
			50	12.1	±	0.1	bc	11.9	±	0.8	bc	ns	10.1	±	0.3	de	10.3	±	0.3	cde	ns
		High	20	12.6	±	0.3	ab	13.4	±	0.4	a	*	11.4	±	0.4	a	11.1	±	0.1	ab	ns
			50	13.2	±	0.3	a	12.7	±	0.2	ab	ns	10.7	±	0.0	bc	10.9	±	0.4	ab	ns
TA (malic acid, %)	2023	Low	20	0.85	±	0.31		0.65	±	0.04		ns	1.15	±	0.03		1.05	±	0.01		**
			50	0.78	±	0.07		0.69	±	0.08		ns	1.16	±	0.05		1.16	±	0.03		ns
		High	20	0.70	±	0.01		0.87	±	0.07		*	1.19	±	0.09		1.17	±	0.07		ns
			50	0.66	±	0.06		0.83	±	0.02		*	1.21	±	0.07		1.16	±	0.04		ns
	2024	Low	20	0.38	±	0.10		0.45	±	0.04		ns	0.98	±	0.08	bc	0.69	±	0.08	c	*
			50	0.54	±	0.00		0.40	±	0.00		***	1.01	±	0.00	bc	0.83	±	0.08	bc	*
		High	20	0.45	±	0.37		0.49	±	0.04		ns	1.14	±	0.07	ab	0.80	±	0.00	bc	***
			50	0.49	±	0.04		0.45	±	0.04		ns	1.36	±	0.50	a	0.80	±	0.00	bc	ns
Dry matter (%)	2023	Low	20	13.7	±	0.6	d	14.2	±	0.3	cd	ns	11.2	±	0.3	abc	10.6	±	0.1	c	*
			50	13.9	±	0.4	d	15.0	±	0.1	bc	*	10.8	±	0.8	bc	11.2	±	0.2	abc	ns
		High	20	14.1	±	0.0	d	14.4	±	0.6	cd	ns	11.3	±	0.6	abc	11.1	±	0.2	abc	ns
			50	15.4	±	0.5	ab	16.2	±	0.5	a	ns	11.8	±	0.1	a	11.6	±	0.3	ab	ns
	2024	Low	20	12.4	±	0.5	d	14.2	±	0.5	abc	*	11.4	±	0.1	d	11.6	±	0.5	cd	ns
			50	13.5	±	0.2	cd	13.6	±	0.3	cbd	ns	11.2	±	0.1	d	11.4	±	0.3	d	ns
		High	20	14.1	±	0.7	abc	15.1	±	0.5	a	ns	12.9	±	0.4	a	12.4	±	0.2	b	ns
			50	15.0	±	0.8	ab	14.2	±	1.6	abc	ns	12.0	±	0.0	bc	12.2	±	0.1	b	*

## Data Availability

The datasets generated and analyzed during the current study are available from the corresponding author upon reasonable request.

## Supplementary Materials

The following supporting information can be downloaded at: https://www.mdpi.com/article/10.3390/plants15010029/s1, Figure S1: Intra-canopy light environment in *Prunus persica* cvs. ‘Platibelle’ and ‘Mirel’ trained to two canopy architectures, a planar two-dimensional (Four-Axis, green) and a three-dimensional (Quad-V, orange) system, during the 2023 and 2024 growing seasons. Photosynthetic photon flux density (PPFD, μmol m^−2^ s^−1^) and relative light availability (%) were measured at representative positions within the lower (L) and upper (H) canopy zones at pit hardening (PH) and harvest (H). Data represent mean values ± SE (n = 4) for each training system and canopy zone. Asterisks denote significant differences between training systems or canopy zones (* *p* < 0.05; ** *p* < 0.01; *** *p* < 0.001; ns = not significant; ANOVA followed by Duncan’s multiple range test); Table S1: Fruit respiration rate (mL CO_2_ kg^−1^ h^−1^) and ethylene production (μL C_2_H_4_ kg^−1^ h^−1^) of *Prunus persica* cvs. ‘Platibelle’ and ‘Mirel’ measured at harvest under two canopy training systems (Quad-V, 3D; and Four-Axis, 2D) during the 2023 and 2024 growing seasons. Data correspond to the same experimental conditions described in Table 1 and are presented for two canopy zones (lower, L; and upper, H) and two thinning levels (20 and 50 leaves per fruit). Values represent mean ± standard deviation (n = 3). Different letters within rows indicate statistically significant differences among treatments according to Duncan’s multiple range test (*p* ≤ 0.05). Where relevant, Student’s *t*-test was applied to compare training systems within each canopy zone. Significance levels: *p* ≤ 0.05; *p* ≤ 0.01; *p* ≤ 0.001; ns = not significant; Figure S2: Pearson correlation matrices between (**a**) physiological parameters and (**b**) fruit quality traits in *Prunus persica* cvs. ‘Platibelle’ and ‘Mirel’ under different fruit loads (20 and 50 leaves per fruit). Data represent the mean values of the 2023 and 2024 seasons. The matrices display Pearson correlation coefficients (*r*) between pairs of variables, with color shading indicating the strength and direction of the relationship (warm tones = positive correlations; cool tones = negative correlations). Significance levels are indicated as *p* < 0.05 (*), *p* < 0.01 (**), and *p* < 0.001 (***); Figure S3: Relationships between canopy structure, light environment, and fruit quality in *Prunus persica* cvs. ‘Platibelle’ and ‘Mirel’ trained to two canopy architectures during the 2023–2024 seasons. Regression analyses were performed to examine the associations between (**a**) fruit firmness and leaf area index (LAI), (**b**) dry matter content and light availability (%) in ‘Platibelle’, (**c**) total orchard productivity (Mt ha^−1^) and LAI and (**d**) fresh fruit weight and light availability (%) in ‘Mirel’. Panel (**e**) presents a scatter plot showing the relationship between LAI and total production for both cultivars combined. Each point represents the mean value of four replicates per training system. Lines indicate significant linear regressions.
