# Supplementary material for: Canopy Design Drives Photosynthetic Performance, Light Environment, and Fruit Quality in Peach (Prunus persica L. Batsch)
Source: Plants (Basel). 2025 Dec 21;15(1):29. doi: 10.3390/plants15010029 (PMC12787653; doi:10.3390/plants15010029)
Supplement: Supplementary file 1 [file plants-15-00029-s001.zip › Figure S1.pptx]

## Slide 1
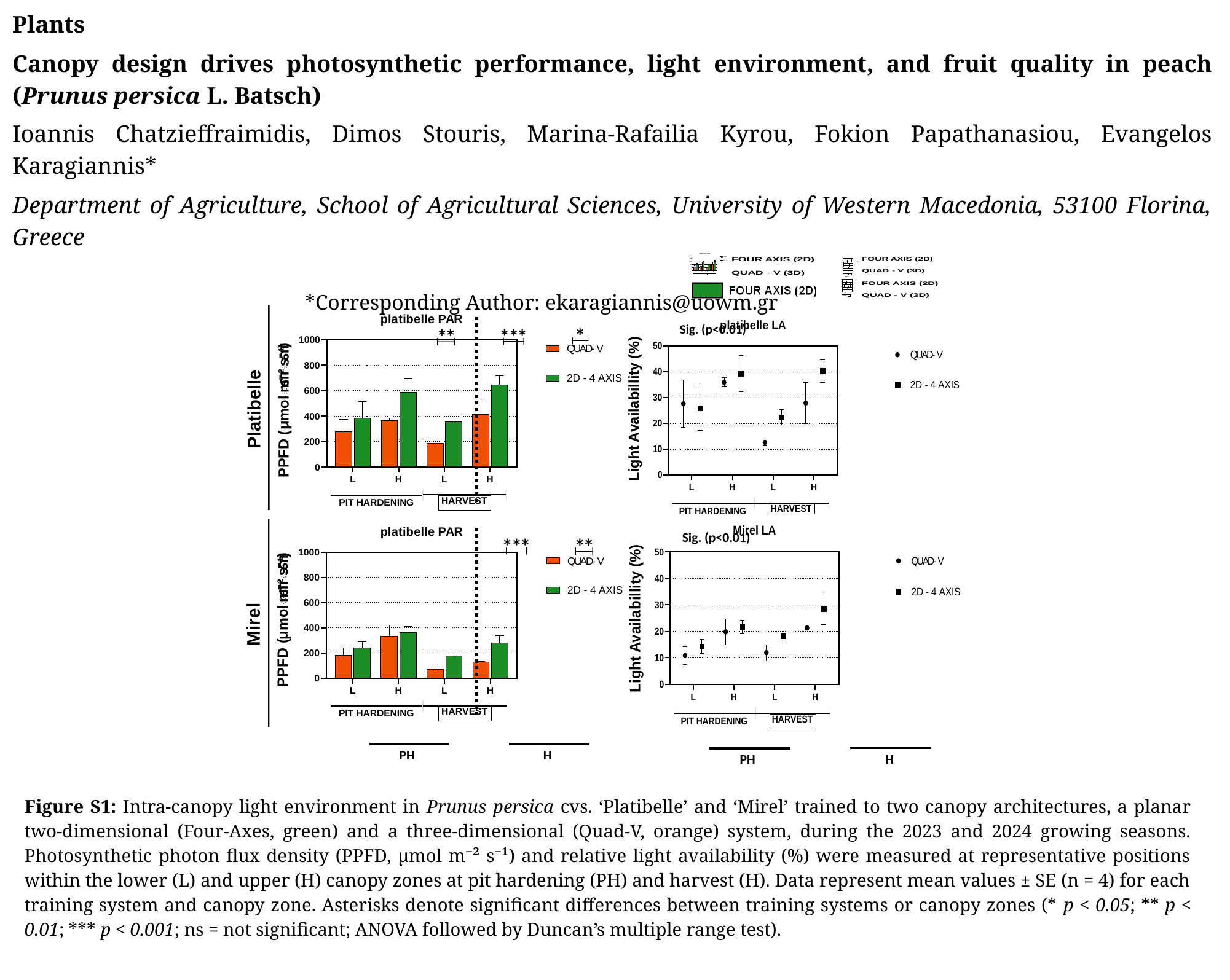

Plants
Canopy design drives photosynthetic performance, light environment, and fruit quality in peach (Prunus persica L. Batsch)
Ioannis Chatzieffraimidis, Dimos Stouris, Marina-Rafailia Kyrou, Fokion Papathanasiou, Evangelos Karagiannis*
Department of Agriculture, School of Agricultural Sciences, University of Western Macedonia, 53100 Florina, Greece
																 *Corresponding Author: ekaragiannis@uowm.gr
Figure S1: Intra-canopy light environment in Prunus persica cvs. ‘Platibelle’ and ‘Mirel’ trained to two canopy architectures, a planar two-dimensional (Four-Axes, green) and a three-dimensional (Quad-V, orange) system, during the 2023 and 2024 growing seasons. Photosynthetic photon flux density (PPFD, μmol m⁻² s⁻¹) and relative light availability (%) were measured at representative positions within the lower (L) and upper (H) canopy zones at pit hardening (PH) and harvest (H). Data represent mean values ± SE (n = 4) for each training system and canopy zone. Asterisks denote significant differences between training systems or canopy zones (* p < 0.05; ** p < 0.01; *** p < 0.001; ns = not significant; ANOVA followed by Duncan’s multiple range test).
