# Supplementary material for: Canopy Design Drives Photosynthetic Performance, Light Environment, and Fruit Quality in Peach (Prunus persica L. Batsch)
Source: Plants (Basel). 2025 Dec 21;15(1):29. doi: 10.3390/plants15010029 (PMC12787653; doi:10.3390/plants15010029)
Supplement: Supplementary file 1 [file plants-15-00029-s001.zip › Figure S2.pptx]

## Slide 1
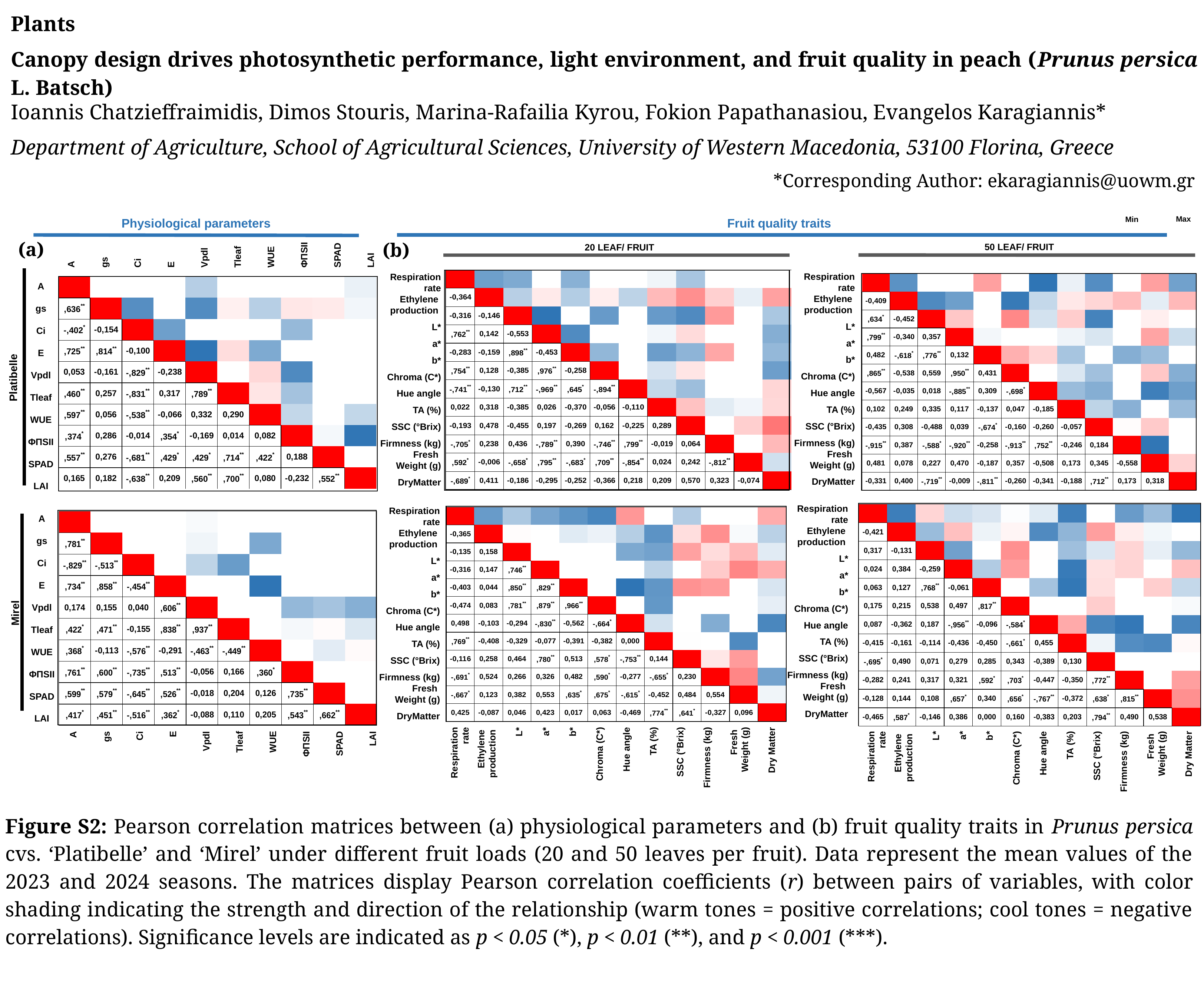

Plants
Canopy design drives photosynthetic performance, light environment, and fruit quality in peach (Prunus persica L. Batsch)
Ioannis Chatzieffraimidis, Dimos Stouris, Marina-Rafailia Kyrou, Fokion Papathanasiou, Evangelos Karagiannis*
Department of Agriculture, School of Agricultural Sciences, University of Western Macedonia, 53100 Florina, Greece
*Corresponding Author: ekaragiannis@uowm.gr
Max
Min
Physiological parameters
Fruit quality traits
A
gs
Ci
E
Vpdl
Tleaf
WUE
ΦΠSII
SPAD
LAI
A
gs
Ci
E
Vpdl
Tleaf
WUE
ΦΠSII
SPAD
LAI
(a)
(b)
50 LEAF/ FRUIT
20 LEAF/ FRUIT
Respiration
 rate
Ethylene
production
L*
a*
b*
Chroma (C*)
Hue angle
TA (%)
SSC (°Brix)
Firmness (kg)
Fresh
Weight (g)
DryMatter
Respiration
 rate
Ethylene
production
L*
a*
b*
Chroma (C*)
Hue angle
TA (%)
SSC (°Brix)
Firmness (kg)
Fresh
Weight (g)
DryMatter
Platibelle
A
gs
Ci
E
Vpdl
Tleaf
WUE
ΦΠSII
SPAD
LAI
A
gs
Ci
E
Vpdl
Tleaf
WUE
ΦΠSII
SPAD
LAI
Respiration
 rate
Ethylene
production
L*
a*
b*
Chroma (C*)
Hue angle
TA (%)
SSC (°Brix)
Firmness (kg)
Fresh
Weight (g)
DryMatter
Respiration
 rate
Ethylene
production
L*
a*
b*
Chroma (C*)
Hue angle
TA (%)
SSC (°Brix)
Firmness (kg)
Fresh
Weight (g)
Dry Matter
Respiration
 rate
Ethylene
production
L*
a*
b*
Chroma (C*)
Hue angle
TA (%)
SSC (°Brix)
Firmness (kg)
Fresh
Weight (g)
DryMatter
Respiration
 rate
Ethylene
production
L*
a*
b*
Chroma (C*)
Hue angle
TA (%)
SSC (°Brix)
Firmness (kg)
Fresh
Weight (g)
Dry Matter
Mirel
Figure S2: Pearson correlation matrices between (a) physiological parameters and (b) fruit quality traits in Prunus persica cvs. ‘Platibelle’ and ‘Mirel’ under different fruit loads (20 and 50 leaves per fruit). Data represent the mean values of the 2023 and 2024 seasons. The matrices display Pearson correlation coefficients (r) between pairs of variables, with color shading indicating the strength and direction of the relationship (warm tones = positive correlations; cool tones = negative correlations). Significance levels are indicated as p < 0.05 (*), p < 0.01 (**), and p < 0.001 (***).
