# Supplementary material for: Canopy Design Drives Photosynthetic Performance, Light Environment, and Fruit Quality in Peach (Prunus persica L. Batsch)
Source: Plants (Basel). 2025 Dec 21;15(1):29. doi: 10.3390/plants15010029 (PMC12787653; doi:10.3390/plants15010029)
Supplement: Supplementary file 1 [file plants-15-00029-s001.zip › Figure S3.pptx]

## Slide 1
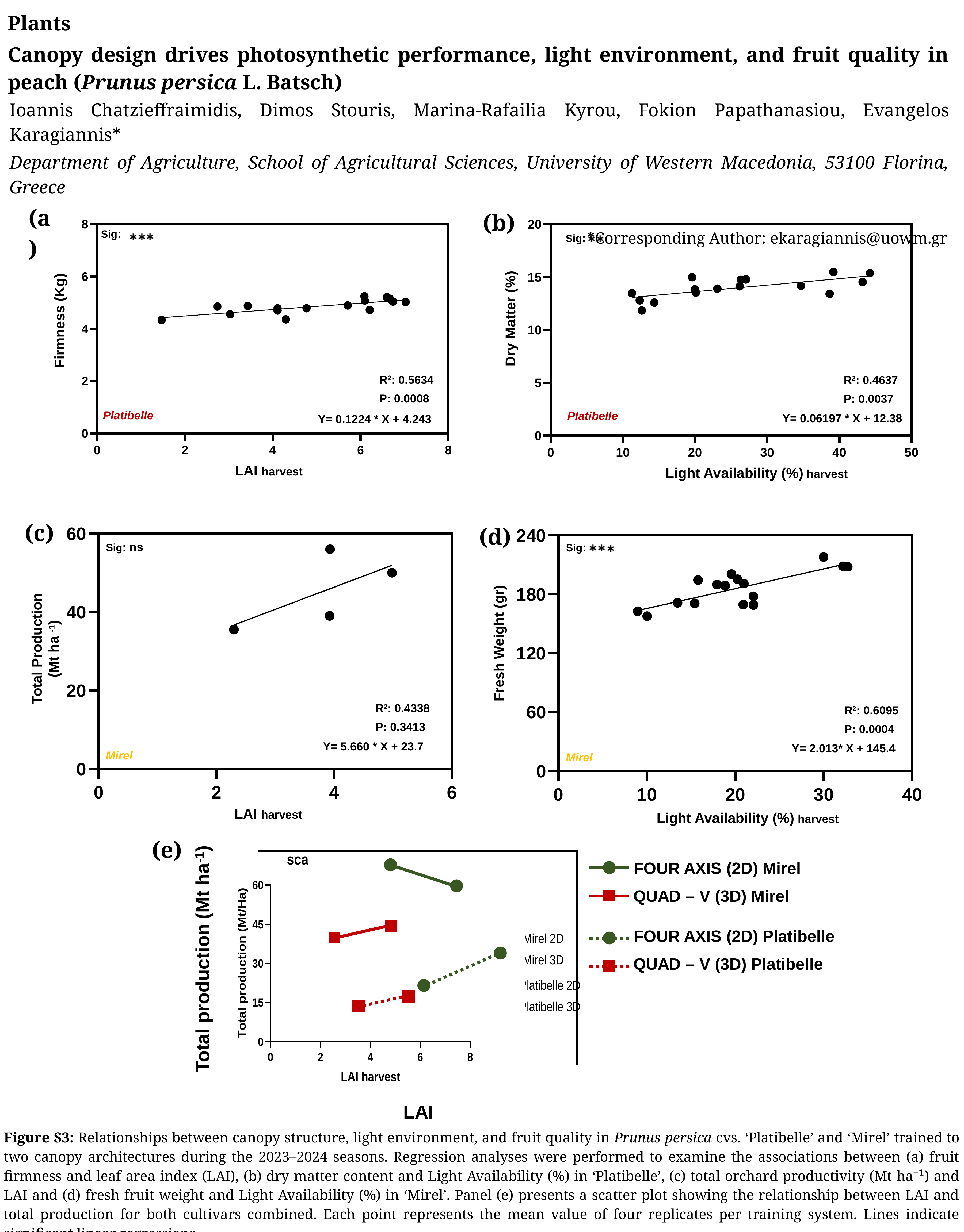

Plants
Canopy design drives photosynthetic performance, light environment, and fruit quality in peach (Prunus persica L. Batsch)
Ioannis Chatzieffraimidis, Dimos Stouris, Marina-Rafailia Kyrou, Fokion Papathanasiou, Evangelos Karagiannis*
Department of Agriculture, School of Agricultural Sciences, University of Western Macedonia, 53100 Florina, Greece
*Corresponding Author: ekaragiannis@uowm.gr
(a)
(b)
Sig:
Firmness (Kg)
R2: 0.5634
P: 0.0008
Platibelle
Y= 0.1224 * X + 4.243
LAI harvest
Dry Matter (%)
Light Availability (%) harvest
Sig:
R2: 0.4637
P: 0.0037
Platibelle
Y= 0.06197 * X + 12.38
(c)
(d)
Total Production
(Mt ha -1)
LAI harvest
Fresh Weight (gr)
Light Availability (%) harvest
Sig: ns
Sig:
R2: 0.4338
R2: 0.6095
P: 0.3413
P: 0.0004
Y= 5.660 * X + 23.7
Y= 2.013* X + 145.4
Mirel
Mirel
(e)
Figure S3: Relationships between canopy structure, light environment, and fruit quality in Prunus persica cvs. ‘Platibelle’ and ‘Mirel’ trained to two canopy architectures during the 2023–2024 seasons. Regression analyses were performed to examine the associations between (a) fruit firmness and leaf area index (LAI), (b) dry matter content and Light Availability (%) in ‘Platibelle’, (c) total orchard productivity (Mt ha⁻¹) and LAI and (d) fresh fruit weight and Light Availability (%) in ‘Mirel’. Panel (e) presents a scatter plot showing the relationship between LAI and total production for both cultivars combined. Each point represents the mean value of four replicates per training system. Lines indicate significant linear regressions.
